# Effectiveness of a decision aid for patients with depression: A randomized controlled trial

**DOI:** 10.1111/hex.12553

**Published:** 2017-03-10

**Authors:** Lilisbeth Perestelo‐Perez, Amado Rivero‐Santana, Juan Antonio Sanchez‐Afonso, Jeanette Perez‐Ramos, Carmen Luisa Castellano‐Fuentes, Karen Sepucha, Pedro Serrano‐Aguilar

**Affiliations:** ^1^ Evaluation Unit of the Canary Islands Health Service (SESCS) Tenerife Spain; ^2^ Health Services Research on Chronic Patients Network (REDISSEC) Tenerife Spain; ^3^ Center for Biomedical Research of the Canary Islands (CIBICAN) Tenerife Spain; ^4^ Canary Islands Foundation of Health Research (FUNCANIS) Tenerife Spain; ^5^ Health Decision Sciences Center (HDSC) Massachusetts General Hospital Boston MA USA

**Keywords:** decision aid, depression, patient involvement (empowerment, self‐management), primary care, randomized controlled trial, shared decision making, Spain

## Abstract

**Background:**

Shared decision making is an important component of patient‐centred care and decision aids are tools designed to support patients' decision making and help patients with depression to make informed choices.

**Objective:**

The study aim was to assess the effectiveness of a web‐based decision aid for patients with unipolar depression.

**Design:**

Randomized controlled trial.

**Setting and participants:**

Adults diagnosed with a major depressive disorder and recruited in primary care centres were included and randomized to the decision aid (n=68) or usual care (n=79).

**Intervention:**

Patients in the decision aid group reviewed the decision aid accompanied by a researcher.

**Outcome measures:**

Knowledge about treatment options, decisional conflict, treatment intention and preference for participation in decision making. We also developed a pilot measure of concordance between patients' goals and concerns about treatment options and their treatment intention.

**Results:**

Intervention significantly improved knowledge (*P*<.001) and decisional conflict (*P*<.001), and no differences were observed in treatment intention, preferences for participation, or concordance. One of the scales developed to measure goals and concerns showed validity issues.

**Conclusion:**

The decision aid “Decision making in depression” is effective improving knowledge of treatment options and reducing decisional conflict of patients with unipolar depression. More research is needed to establish a valid and reliable measure of concordance between patients' goals and concerns regarding pharmacological and psychological treatment, and the choice made.

## 1 INTRODUCTION

In the last decade, the active participation of patients in the decision making process regarding their health care has been increasingly advocated.^1^, ^2^, ^3^ One of the conceptual models proposed within this new patient‐centred perspective of health care is the shared decision making (SDM) model. SDM is a process of joint deliberation and collaboration between the health professional and the patient in order to reach a consensus about treatment or diagnostic decisions. In this dyadic interaction, health professionals offer technical information about the disease, the benefits, and risks of the available therapeutic or diagnostic options, whereas patients provide information about their beliefs, concerns, values, and preferences about the consequences of those options.^4^, ^5^, ^6^ SDM is especially relevant when the scientific evidence about the effectiveness or safety of available treatments is scarce, or when they show a similar balance between benefits and risks.

Patients decision aids (DAs) are tools designed to promote and facilitate SDM and help patients to make informed choices.^7^, ^8^, ^9^ These materials are developed in different formats (paper and pencil instruments, videos, audio‐guided workbooks, web‐based tools, interactive software, etc.) and can be used alone by the patient or in interaction with the health professional. DAs include explanations about treatment options, describing the benefits and harms based on the scientific evidence. They also encourage patients to think about their own values and preferences regarding the benefits and risks of the different treatment options, and how they could influence their lives and well‐being.^10^, ^11^

Recent systematic reviews show that DAs are effective in improving patients' knowledge about available treatments, risk perceptions, and their decisional conflict (uncertainty about the course of action to take). They also have shown to reduce the proportion of people who were passive in decision making or who remained undecided after deliberation. Mixed or inconclusive results have been found on other outcomes such as satisfaction with the decision making process, adherence to treatment or consultation time.^12^, ^13^, ^14^

In the field of mental health, although some studies have pointed out some psychiatrists' concerns about the capacity of patients to engage in SDM, due to a lower awareness of the disease or reduced cognitive abilities,^15^, ^16^ research has shown that most of these patients can understand treatment choices and make rational decisions.^17^, ^18^ In the specific area of depressive disorders, results show that a majority of these patients are interested in receiving information about their illness and participating in SDM,^19^, ^20^, ^21^, ^22^ and perceive a lesser involvement in decisions than they desire.^23^, ^24^, ^25^ This perception is confirmed by studies that objectively assessed SDM facilitation in practice by psychiatrists^26^ or primary care physicians.^27^, ^28^ However, despite this demand there have been very few studies that have assessed the effectiveness of DAs or other decision support interventions in the field of depressive disorders. To our knowledge, only three randomized trials have implemented a DA for patients with depression;^29^, ^30^, ^31^ one of them also included an intense 6‐month training for physicians in the concepts and practice of SDM.^29^ Results showed significant differences favouring intervention groups in decisional conflict,^30^, ^31^ preparation for decision making and preference for participation,^29^ and several domains of satisfaction,^31^ whereas mixed results were observed on knowledge^30^, ^31^ and patients' perception of their involvement facilitated by the doctor.^29^, ^30^ No significant effects were observed on the severity of depression or treatment adherence.^29^, ^31^

Our research group has developed a Web Platform (PyDeSalud.com) to promote and facilitate citizens' empowerment and engagement in the decisions concerning their health.^32^ It offers different resources, including DAs, for several highly prevalent health conditions. The present study aims to add new evidence about the effectiveness of DAs for patients with depression, assessing the effect of a web‐based DA on several patients' decisional outcomes. As the primary objective, we hypothesized that the DA would decrease patients' decisional conflict. Secondarily, we expected that the intervention would improve their knowledge of treatment options, increase the number of patients who are sure about the treatment choice and of those who prefer to share this decision with their health‐care provider, instead of playing a passive role. In addition, we developed a measure of concordance between patients' goals/concerns about treatment options and their intention to choose a particular treatment, because this outcome has been recently proposed as a quality criterion of the decisional process;^33^ we expected that the intervention would increase the number of participants who make a concordant choice.

## 2 METHOD

### 2.1 Study design and participants

A randomized controlled trial was performed in 13 primary care centres in Tenerife (Spain). Patients were eligible if they were 18 or older, had a major depressive disorder according to the ICD‐10^34^ or the DSM‐IV‐TR^35^ diagnostic criteria, and spoke Spanish language. Between June 2014 and June 2015, 26 primary care professionals presented the study to their (consecutive) eligible patients, and those who were interested in participate were contacted by telephone by a researcher. They were informed in detail about the study aims and procedures, and definitively decided about their participation. Those who consented were given an appointment in our research centre. A simple randomization schedule (ratio 1:1) to intervention (web‐based DA) or control group (usual care) was performed by an independent researcher, by means of a computer software. Both physicians and the researcher who informed and recruited the patients were unaware of patients' allocation.

The Scientific and Ethics Committee of the University Hospital Nuestra Señora de la Candelaria approved the study protocol. The study was performed in accordance with Good Clinical Practice standards, applicable local regulatory requirements, and the recommendations of the Declaration of Helsinki.

### 2.2 Intervention and procedure

The Web Platform PyDeSalud.com is a medical website developed to improve citizens' knowledge of highly prevalent diseases such as diabetes, depression, or cancer, and promote their active participation in health‐care decisions.^32^

It contains three modules of information services: (i) narratives of real patients' experiences, (ii) DAs, and (iii) research needs from the perspective of patients that have to be fulfilled in the scientific literature. The module “Decision making in depression” is a DA for patients with unipolar depression; it was developed according to recommended methods,^36^ including patients' and professionals' perspectives by means of focus groups and individual interviews, in an iterative process. Through the DA, the patient can learn about symptoms, types of depression, treatment options and its characteristics (probabilities of response, remission and relapse, adverse effects, and other problems), information resources and support, etc. It includes a values clarification exercise in which patients have to rate the importance attributed or concerns related to different aspects of treatments. At the time of the study, the DA was not publically accessible.

Participants in the study came to our research centre and signed informed consent. Intervention patients completed the baseline questionnaires (outcome measures were not assessed at baseline) and then reviewed the DA in a single session, accompanied by a researcher who gave support in navigation and clarified patients' questions when necessary. Participants took the time they considered necessary to review the DA. Immediately after, outcome measures were assessed.

Control participants filled the questionnaires assessing all the study variables. They were told that the study aimed to investigate patients' attitudes about the participation in the decision‐making process about their care, but they were not informed about the assessment of the DA until they finished their participation.

### 2.3 Measures

#### 2.3.1 Baseline

Socio‐demographic and clinical data are as follows: age, sex, education, marital and work status, illness duration, whether the patients was taking antidepressants and had experienced adverse effects, and type of health care (only public vs public/private). Severity of depression was assessed with the Spanish version of the Beck Depression Inventory (BDI‐II).^37^

#### 2.3.2 Post‐intervention

##### Primary outcome

*Decisional conflict*: We used the Spanish version of the *Decisional Conflict Scale* (DCS)^38^, ^39^ that evaluates the level of patients' uncertainty when they are confronted to a medical decision. The scale includes 16 items and five subscales: feeling informed, clear values about benefits and risks, support, uncertainty, and effective decision. Items are scored from 0 (strongly agree) to 4 points (strongly disagree), with higher scores indicating higher decisional conflict. The total score is transformed to a 0‐100 scale. In a previous study with Spanish patients diagnosed with type 2 diabetes, the total scale showed a Cronbach's alpha of .90.^40^ We calculated that, assuming equal variances in the intervention and control groups, 126 patients would be needed to detect a moderate effect size (standardized mean difference=0.5), with a confidence level of 95% and a power of 80%.

##### Secondary outcomes

*Knowledge of treatment options* was assessed with an eight‐item scale developed by the authors. Items' content referred to aspects like adverse effects of antidepressants, continuation pharmacotherapy, or time to improve with psychotherapy. Five items had a response format of “true/false/I don't know” and the remaining three had a multiple‐choice format with four response options. The number of correct responses represents the total score.

*Treatment intention*: Patients were asked “If you had to choose a treatment right now, what treatment would you choose?,” with four response alternatives: (i) antidepressant drugs, (ii) psychological treatment, (iii) combined pharmacological and psychological treatment, and (iv) “I'm not sure.” For the aim of the analysis, we collapsed the three former options into a single category, but we also explore whether there were significant differences between study groups in the type of treatment chosen.

*Decisional control preferences*: We applied the item of the Control Preference Scale^41^ that assessed patients' preferred level of involvement in making a decision about treatment. It consists of five statements that range from a completely passive role (only the doctor makes the decision) to a completely active role (only the patient makes the decision). In the analysis, the first two and the last two response levels were respectively collapsed into one, resulting in three categories: passive, shared, and active roles.

*Goals and concerns*: Patients were asked four questions about their goals and concerns related to pharmacological and psychological treatments (Table 1), to be answered in a 0‐10 scale (from “not important at all” to “extremely important” in the case of goals, and from “nothing” to “very much” in the case of concerns). We then pooled the scores of the two items about psychotherapy on one side and the two items about pharmacotherapy on the other, resulting in two separate scales ranging 0‐20 and labelled “Attitude towards psychotherapy” (ATP) and “Attitude towards antidepressants” (ATA), respectively, with higher scores representing a more positive attitude. These two scales, along with the responses to the question about what treatment patients would choose, were used to develop a measure of concordance (see the 2.4 section).

**Table 1:** Scales of goals and concerns, and criteria for defining concordance

*Attitude towards psychotherapy* (*ATP*) (*range 0‐20*)		
How important is for you to learn coping strategies to modify your negative thoughts and inappropriate behaviors?		
How much are you worried about spending time and effort performing the activities of the psychotherapy? (reversed)		
*Attitude towards antidepressants* (*ATA*) (*range 0‐20*)		
How important is for you to avoid the adverse effects of antidepressant drugs? (reversed)		
How much are you worried about taking antidepressants drugs? (reversed)		
*Treatment choice*	*Concordance criteria* (*midpoint*)	*Concordance criteria* (*means*)
Only pharmacotherapy	ATP≤10 and ATA>10	ATP≤18 and ATA>7
Only psychotherapy	ATP>10 and ATA≤10	ATP>18 and ATA≤7
Combined therapy	ATP>10 and ATA>10	ATP>18 and ATA>7

### 2.4 Statistical analysis

Analyses were performed with the SPSS 22.0 software. *T*‐test and Chi‐squared test, for continuous and categorical variables, respectively, were used to analyse the effect of the intervention from a univariate perspective. Then, several multiple linear regression models were performed with each one of the continuous outcome measures (decisional conflict and its subscales, knowledge) as dependent variables, and the experimental group as the independent variable. For exploratory aims, the following socio‐demographic and clinical confounders were included in the model: age, sex, education level, depression severity and duration, being taking antidepressants, having experienced antidepressants adverse effects, and receiving private health services.

In addition, we developed a measure of concordance between patients' goals/concerns and their treatment intention. There is not a standardized method to measure this concordance.^33^, ^42^ We applied a “simple match” approach in which, after excluding those patients who answered “not sure” to the question about what treatment they would choose, the remaining patients' choices were classified as concordant or not depending on whether their scores on ATP and ATA were above or under the scales' midpoint (that is, >10 or ≤10). For instance, a patient who would choose only pharmacotherapy is classified as concordant if she/he scores above the midpoint in ATA and under that threshold in ATP, and as non‐concordant in any other case (see Table 1). Then, we compared the number of patients who made a concordant choice in the intervention and control groups, respectively, both from a univariate (Chi‐squared test) and multivariate (logistic regression with concordance as dependent variable) perspective. Finally, we compared the results to those obtained by using the scales' means as thresholds for defining concordance, instead of the midpoint. Statistical analyses were performed by a researcher blinded to participants' allocation.

In the intervention group, a range of 4‐8 patients, depending on the outcome measure, missed or refused (due to fatigue) to complete some scales or subscales; we adopted a conservative intention‐to‐treat (ITT) approach, imputing those values with the completers' observed mean plus (for decisional conflict) or minus (for knowledge) a standard deviation, therefore supposing a worse scenario for our hypothesis (lower knowledge and/or higher decisional conflict than the average participant). Discrepancies between ITT and completers analyses will be reported.

## 3 RESULTS

**Figure 1:**
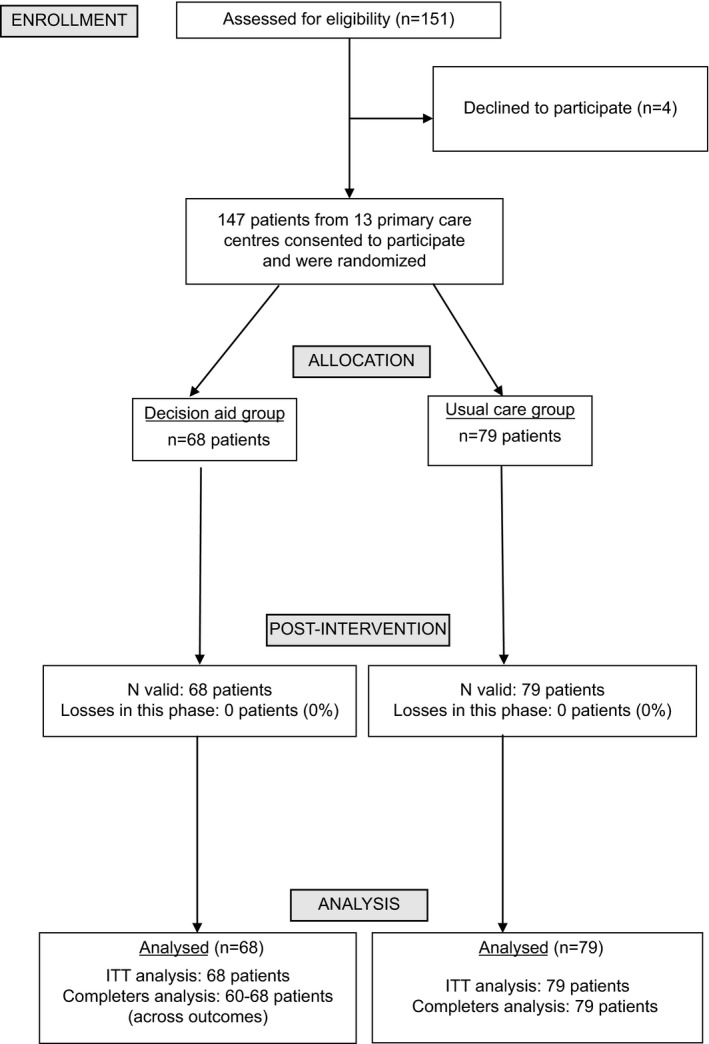
CONSORT flow diagram of participants through the study

The Figure 1 shows the flow of patients through the trial. Physicians informed about the study to 151 patients, from whom four decline participation at that moment. All the remaining 147 patients consented to participate when the research team contacted them. Baseline data for intervention and control groups are shown in Table 2. Sixty‐eight and 79 were randomized to the intervention (DA) and control groups, respectively. Mean age was 51.3 (SD=12.9), the percentage of women was 77.6%, and 44.2% of participants had secondary education or more. Onset of depressive symptoms was 12.5 (SD=13.1) years ago on average, and the mean BDI‐II score was 20.0 (SD=12.4), that represents a moderate level of depression. Eighty‐five percentage of patients were on antidepressant medication, 76.2% were taking other psychoactive drug (mostly benzodiazepines), and only three (2%) were receiving psychotherapy. The DCS showed a Cronbach's alpha of .88.

### 3.1 Acceptability of the DA

Sixty‐one intervention patients offered data about DA acceptability. Most of them agreed or strongly agreed (score of 4 or 5 in a 5‐point scale) that the DA was useful (91.8%), easy to navigate (88.5%), visually appealing (88.5%), entertaining (90.1%), and that they would use it for choosing treatment (80.3%). In a 7‐point scale, the percentage of patients who scored 6 or 7 on the different dimensions assessed was: 88.5% thought that the information was clear, 78.8% would recommend it to a friend, 78.7% and 72.1% stated that they had learned new things about benefits and risk of treatments, respectively, and a 70.5% would ask their physician about these topics they had learned. However, 85.2% thought that the quantity of information was too much.

**Table 2:** Sociodemographic and clinical data at baseline

	DA group (n=68)	Control group (n=79)
Age	51.8 (11.5)	50.9 (14.0)
Women	49 (72.1%)	65 (82.3%)
Education		
No formal education	16 (23.5%)	11 (13.9%)
Primary studies	22 (32.4%)	33 (41.8%)
Secondary studies	22 (32.4%)	19 (24.1%)
University studies	8 (11.8%)	16 (20.3%)
Marital status		
Single	8 (11.8%)	21 (26.6%)
Married	36 (52.9%)	35 (44.3%)
Divorced	19 (27.9%)	17 (21.5%)
Widow	5 (7.4%)	6 (7.6%)
Laboral status		
Employed	22 (32.4%)	22 (28.9%)
Unemployed	13 (19.1%)	13 (17.1%)
Sick leave	12 (17.6%)	13 (17.1%)
Retired	13 (19.1%)	19 (25%)
Housework	8 (11.8%)	9 (11.8%)
Time since onset of depressive symptoms (years)	13.6 (13.7)	11.6 (12.5)
Depression severity (BDI‐II)	18.4 (13.6)	21.2 (11.3)
Taking antidepressant medication	58 (85.3%)	67 (84.8%)
Having experienced antidepressants AEs	32 (50.0%)	30 (40.5%)
Taking other psychoactive medication	54 (79.4%)	58 (73.4%)
Receiving psychotherapy	1 (1.5%)	2 (2.5%)
Receiving private health care	14 (20.6%)	17 (21.5%)

AEs, adverse effects; BDI‐II, Beck Depression Inventory; DA, decision aid.

Data are means (SD) and n (%) for continuous and categorical variables respectively.

### 3.2 Effect of the intervention

Table 3 shows the results of the univariate analyses on the outcome measures assessed, and Table 4 shows the results of the linear regression models, controlling for socio‐demographic and clinical confounders (unstandardized betas are shown). The intervention significantly decreased decisional conflict total score (*B*=−9.98, *P*<.001), and the subscales “informed” and “effectiveness”. The “support” subscale only yielded a significant difference in the completer's analysis, also favouring the DA (*P*=.044). Among the remaining independent variables, receiving private health services was significantly associated with improvements in DCS total score and its subscales, excepting “informed” and “effectiveness”, whereas having university education (vs primary education), and less time since onset of symptoms significantly predicted scores on the feeling of being informed. Having experienced antidepressants adverse effects related to less conflict on the “values” subscale.

**Table 3:** Effect of the intervention (univariate results)

	DA group (n=68)	Control group (n=79)	*P* a
DCS total score (0‐100)	23.65 (15.89)	35.40 (15.30)	<.001
Informed	55.47 (32.57)	74.26 (27.15)	<.001
Values	17.71 (12.90)	18.67 (15.34)	.690
Support	16.53 (21.27)	23.21 (15.49)	.033
Uncertainty	19.26 (24.65)	34.28 (26.85)	.001
Effectiveness	14.34 (21.87)	28.80 (22.07)	<.001
Knowledge (0‐8)	6.89 (1.25)	4.63 (1.48)	<.001
Intention to choose			
Sure^b^	62 (96.9%)	69 (87.3%)	.041
Not sure	2 (3.1%)	10 (12.7%)	
Medication	5 (7.8%)	7 (8.9%)	.185
Psychotherapy	15 (23.4%)	13 (16.5%)	
Combined	42 (65.6%)	49 (62.0%)	
Not sure	2 (3.1%)	10 (12.7%)	
Decisional control preference			
Passive	43 (54.4%)	35 (54.7%)	.149
Shared	28 (43.8%)	29 (36.7%)	
Active	1 (1.6%)	7 (8.9%)	
Patients who made a concordant choice^c^	23 (37.1%)	27 (39.1%)	.811

DA, decision aid; DCS, Decisional Conflict Scale.

a *P*‐values from *t* test, for continuous variables (mean, SD), and χ^2^ for categorical variables (n, %).

b Patients who stated an intention to choose medication, psychotherapy or combined therapy.

c Patients who answered “not sure” to the question about treatment choice were excluded (n=12).

**Table 4:** Results of the lineal regression models on knowledge, decisional conflict and its subscales^a^

	DCS	Informed	Values	Support	Uncertainty	Effectiveness	Knowledge
DA intervention	−9.98 (−15.6, −4.38)^d^	−20.8 (−30.9, −10.8)^d^	−0.52 (−5.41, 4.37)	−5.01 (−11.6, 1.63)	−11.9 (−21.3, −2.48)^d^	−11.3 (−19.4, −3.14)^d^	2.33 (1.85, 2.81)^d^
Age	0.10 (−0.14, 0.34)	0.05 (−0.39, 0.48)	0.15 (−0.06, 0.36)	−0.04 (−0.32, 0.25)	0.17 (−0.24, 0.58)	0.20 (−0.16, 0.55)	0.01 (−0.01, 0.03)
Sex	2.21 (−4.69, 9.10)	3.46 (−8.91, 15.8)	−2.71 (−8.72, 3.31)	0.36 (−7.81, 8.52)	4.67 (−6.92, 16.3)	3.20 (−6.80, 13.2)	−0.17 (−0.76, 0.42)
No formal education^b^	−0.43 (−8.39, 7.53)	3.16 (−11.1, 17.4)	−1.69 (−8.63, 5.25)	4.76 (−4.66, 14.2)	−5.40 (−18.8, 7.98)	0.50 (−11.1, 12.0)	−0.51 (−1.19, 0.17)
Secondary education^b^	−4.20 (−11.6, 3.16)	−8.62 (−21.8, 4.58)	0.34 (−6.08, 6.76)	−1.95 (−10.7, 6.77)	−5.33 (−17.7, 7.03)	−4.52 (−15.2, 6.15)	0.22 (−0.41, 0.85)
University education^b^	−6.46 (−14.6, 1.73)	−20.8 (−35.5, −6.17)^d^	−3.17 (−10.3, 3.97)	1.97 (−7.72, 11.7)	−6.09 (−19.8, 7.65)	−2.93 (−14.8, 8.93)	0.61 (−0.09, 1.31)
Depression severity^c^	0.12 (−0.11, 0.35)	0.17 (−0.24, 0.58)	0.16 (−0.04, 0.36)	−0.01 (−0.28, 0.26)	0.22 (−0.17, 0.60)	0.08 (−0.25, 0.41)	0.01 (−0.01, 0.03)
Time since onset	0.22 (−0.03, 0.46)	0.61 (0.17, 1.05)^d^	0.12 (−0.09, 0.34)	0.09 (−0.20, 0.38)	0.31 (−0.10, 0.72)	0.07 (−0.29, 0.42)	−0.03 (−0.05, −0.01)^d^
Taking antidepressants	−3.06 (−12.1, 6.00)	−11.1 (−27.4, 5.13)	−0.06 (−7.96, 7.85)	0.46 (−10.3, 11.2)	−2.36 (−17.6, 12.9)	−3.04 (−16.2, 10.1)	−0.65 (−1.43, 0.13)
Having experienced antidepressants AEs	1.17 (−4.34, 6.67)	4.50 (−5.37, 14.4)	−5.27 (−10.1, −0.47)^d^	4.61 (−1.91, 11.1)	3.36 (−5.89, 12.6)	0.28 (−7.71, 8.26)	−0.20 (−0.68, 0.27)
Receiving private health services	−9.13 (−16.0, −2.28)^d^	−11.1 (−23.4, 1.20)	−6.66 (−12.6, −0.69)^d^	−8.17 (−16.3, −0.06)^d^	−12.9 (−24.4, −1.40)^d^	−8.21 (−18.1, 1.72)	0.68 (0.09, 1.27)^d^

AEs, adverse effects; DA, decision aid; DCS, Decisional Conflict Scale.

a Data are unstandardized beta values (95% confidence interval). For DCS total score and subscales (score range 0‐100) negative values indicate a better result for the intervention group, whereas the opposite applied to Knowledge (range 0‐8).

b Reference is primary studies.

c Assessed with the Beck Depression Inventory II (BDI‐II).

d Significant results.

In the case of patients' knowledge of treatment options, results showed a significant effect favouring the DA intervention (*B*=2.33, *P*<.001). In addition, those with less time since depression onset and those receiving private (vs only public) health services scored significantly higher. Having university education (compared to those with primary education) significantly related to higher knowledge only in the completers' analysis.

Data about the patients' treatment intention after viewing the DA are shown in Table 3. As a whole, most participants (62%) preferred the combined treatment; in the intervention group, there were more patients that would choose psychotherapy and less that were not sure about the decision, but the difference was not significant (*P*=.185). However, when we compared the rate of patients who preferred a particular treatment (medication, psychotherapy, and combined treatment collapsed into one category) to those who were not sure, the difference favouring the intervention group was significant (*P*=.041). There were not significant differences in control preferences (Table 3).

When using the midpoints of the ATP and ATA scales as thresholds to define concordance between patients' goals/concerns and treatment intention, a total of 50 patients (38.2%, after excluding those who were not sure about treatment choice) were classified as making a concordant choice. There were not significant differences between intervention and control groups in the univariate (37.1% vs 39.1%, *P*=.881) or the multivariate analysis (OR=0.50, *P*=.124). In the latter, two variables significantly predicted concordance: lower severity assessed by the BDI‐II (OR=0.96, *P*=.017) and having not experienced adverse effects of antidepressants (OR=0.41, *P*=.045). The rate of concordant decisions was higher in the group that would choose psychotherapy (25/28, 89.3%) than in the “combined therapy” group (25/91, 27.5%) and the “only pharmacotherapy” group (0/12, 0%) (*P*<.001 for the between‐group difference). When the mean scores of ATP and ATA scales (18 and 7 respectively) were used instead of their midpoints to calculate concordance, the rate of patients who made concordant decisions decreased (31.3%) and the remaining analyses yielded similar results.

## 4 DISCUSSION AND CONCLUSION

### 4.1 Discussion

Our results confirm previous evidence on the effectiveness of DAs in decreasing patients' decisional conflict and increasing their knowledge.^12^, ^30^, ^31^ Furthermore, the effects observed were more than two‐fold greater than the average effect for other diseases as a whole, a 13% in decisional conflict (vs 5%) and 29% of improvement in knowledge (vs 14%).^12^ This could be due in part to the relatively low educational level of the sample included (18% had no formal education and 37% only primary studies), since it has been observed that disadvantaged or less knowledgeable populations obtain more benefit from decision support interventions.^43^, ^44^ In addition, the high mean score observed in the DCS subscale “informed” (74 in a 0‐100 scale in the control group, that is, a high conflict) favours a potential strong improvement; however, even after the application of the DA the intervention group showed a considerable level of feeling uninformed (56 points). It is possible that viewing the DA, or simply being asked about these issues, makes patients to become aware of their poor knowledge of treatment options, especially about the nature of psychotherapy and its characteristics; very few patients had some experience with psychological treatment (in Spain, public resources for psychotherapy services are limited, and waiting lists are long) and indeed the two knowledge items that exclusively asked about psychotherapy obtained the lowest rates of correct answers, 35% and 50%, respectively. On the other side, results show that receiving private health care was a significant independent predictor of better knowledge and less decisional conflict, which could reflect the lesser time constrains or a more personalized attention in private practices.

In line with the results obtained on decisional conflict, significantly less patients in the intervention group were unsure about the treatment choice. Regarding the specific treatment, there were no significant differences in patients' choices. Most of them (63%) would choose the combined therapy, 19% preferred only psychotherapy, and 8% would choose only medication. The preference for psychotherapy vs medication is in line with previous findings,^45^, ^46^, ^47^, ^48^ whereas mixed results have been observed about the preference for the combined treatment.^45^, ^47^, ^49^ The importance of matching the preferred and received treatment in depression is highlighted by studies that have found better health outcomes and/or lower attrition in those patients who underwent their preferred treatment,^45^, ^50^, ^51^ although former studies had obtained null results.^52^, ^53^ But besides these potential beneficial effects on health outcomes, the concordance between patients' preferences and their treatment choices represents itself a measure of decision quality,^7^, ^53^, ^54^ since the theoretical foundations of SDM rest on a normative rationalistic ideal of decision making.^54^

There is not a commonly agreed‐on method to measure this concordance and different proposals have been published, from “simple match” approaches to more sophisticated techniques like conjoint analysis or regression models, that empirically assessed the associations of patients' values regarding specific characteristics of available options on one side, with their treatment choices on the other.^42^, ^55^, ^56^, ^57^ We carried out a pilot assessment of a measure of concordance developing two simple scales to assess patients' goals and concerns about pharmacological and psychological treatments. We obtained a much lower rate of concordant decisions (38%) than studies in the field of breast cancer (77%‐89%),^56^, ^57^, ^58^ osteoarthritis (73%),^59^ or herniated disc (78%),^60^ but we cannot establish whether these differences are due to the method used, the health condition, or other factors. We performed some validity analyses of the two goals/concerns scales (associations with treatment choice, having experienced adverse effects of antidepressants, illness severity, and duration; data not shown), and results were not optimal, especially for the ATA; for instance, a negative attitude represented by a low score does not imply at all a refusal to take them (in the group that would choose only pharmacotherapy, 9 out of 12 patients scored under the midpoint of the scale, that is, stated a concern about taking antidepressants). Assuming that participants make rational choices, these data suggest that there are relevant features of pharmacological and psychological treatments that have not been assessed in the goals/concern scales.

The main methodological limitation of this study is the absence of baseline assessment. Since all the assessments and the intervention had to be applied in one continue session in our research centre, we did not want to overload intervention participants, and we also wanted to avoid memory effects in the case of the knowledge measure (being tested just before reviewing the DA could reinforced patients' learning of the aspects evaluated). Randomization is expected to overcome this limitation, and indeed we tried to minimize the likelihood of allocation bias by performing the randomization blinded to the recruitment of participants and vice versa. However, it resulted in a quite unbalanced distribution of patients to the intervention and control groups (43.3%/56.7%, respectively, *P*=.05). However, there were not significant differences between groups in baseline socio‐demographic and clinical variables, but reviewing the baseline distributions of patients' characteristics we observed that although the difference is non‐significant, control group included almost twice as many participants with university degree and half with no formal education. Taking into account the significant positive relationship between education level and knowledge found in previous studies on other diseases^61^, ^62^, ^63^, ^64^, ^65^, ^66^, ^67^, ^68^ (replicated in our completers analysis; in addition, patients with university degree also outperformed no formally educated patients in the variable “feeling informed”), this could suggest that allocation imbalance might have been in fact more prejudicial for the intervention group, in terms of the outcomes evaluated.

A second limitation is that blinding of participants is difficult in these interventions, and therefore, a “novelty” or “attention” effect cannot be ruled out. Third, DA was administrated by a researcher instead of the health‐care provider, which would not represent the usual practice; related to this, a previous study that compared the two procedures in type 2 diabetes patients showed a better result in the latter case;^69^ if this result is extensive to other medical conditions, the significant effects obtained in this study could be considered as a worst‐case scenario. Finally, we could not register whether patients actually made changes in their treatment after study completion (instead, we measured the treatment intention, that has been shown to be a significant predictor of actual choices)^70^, ^71^ or tried to obtained more information about treatments' characteristics. However, despite these limitations, we think that this study adds relevant evidence about the effect of DAs on two fundamental variables of the decisional process (decisional conflict and knowledge) in the treatment of depression in primary care, a field in which this evidence, well established for somatic diseases,^12^, ^13^, ^14^ is scarce.

### 4.2 Conclusion

The decision aid “Decision making in depression” is effective improving decisional conflict and knowledge of treatment options of patients with unipolar depression. More research is needed to establish a valid and reliable measure of concordance between patients' goals and concerns regarding pharmacological and psychological treatment, and the choice made.

## CONFLICT OF INTEREST

The authors have no conflict of interest to declare.
